# Supplementary figures and images for: Transcriptomic comparison of two selective retinal cell ablation paradigms in zebrafish reveals shared and cell-specific regenerative responses
Source: PLoS Genet. 2023 Oct 11;19(10):e1010905. doi: 10.1371/journal.pgen.1010905 (PMC10593236; doi:10.1371/journal.pgen.1010905)

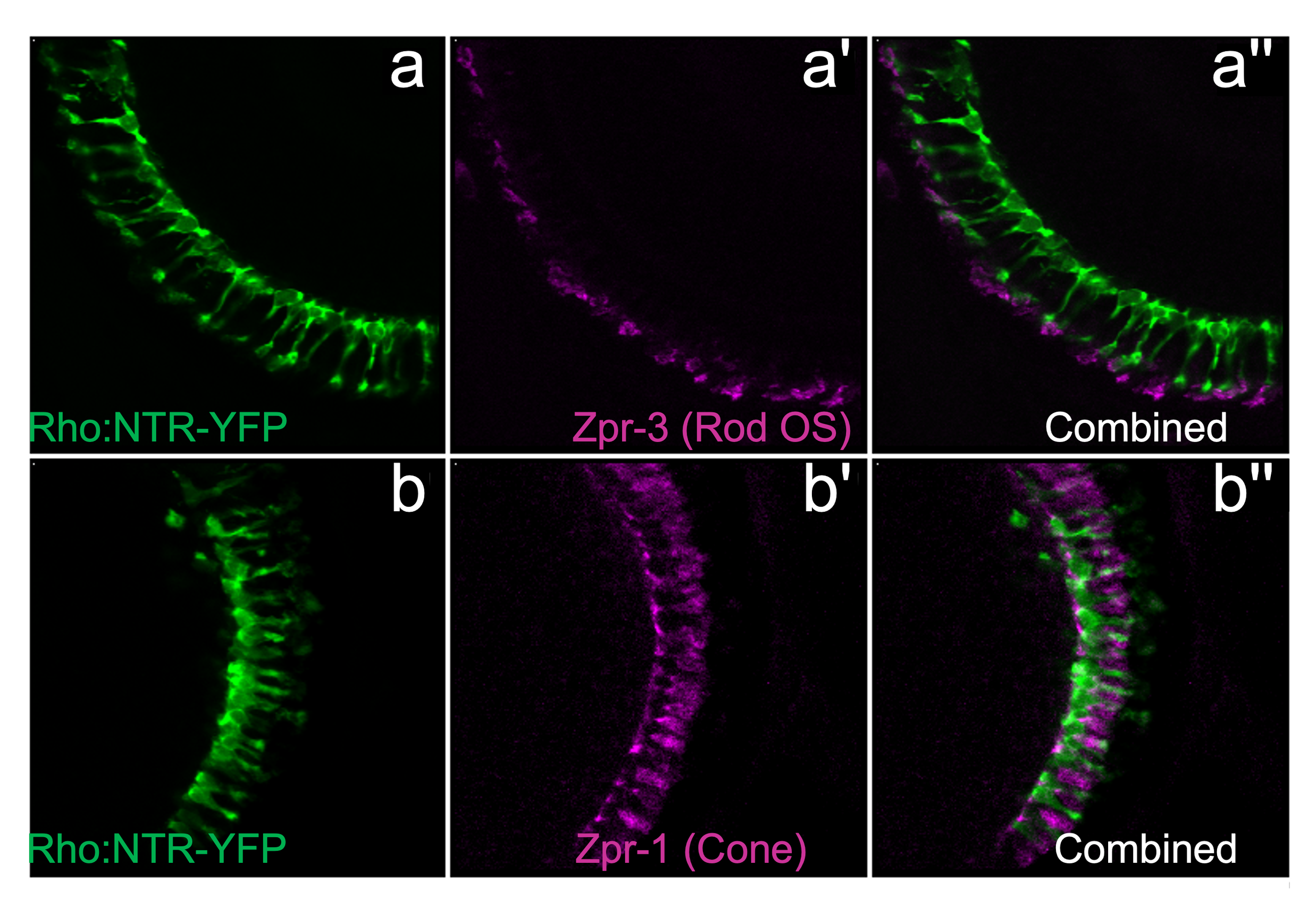

Supplement: S1 Fig — (a-a”) Histological staining in the NTR-rod model showing transgenic Rho:YFP-NTR expression (green) along with antibody staining for rod outer segment marker Zpr-3 (purple). (b-b”) Histological staining in the NTR-rod model showing transgenic Rho:YFP-NTR expression (green) along with antibody staining for cone marker Zpr-1 (purple). (TIF) [file pgen.1010905.s001.tif]

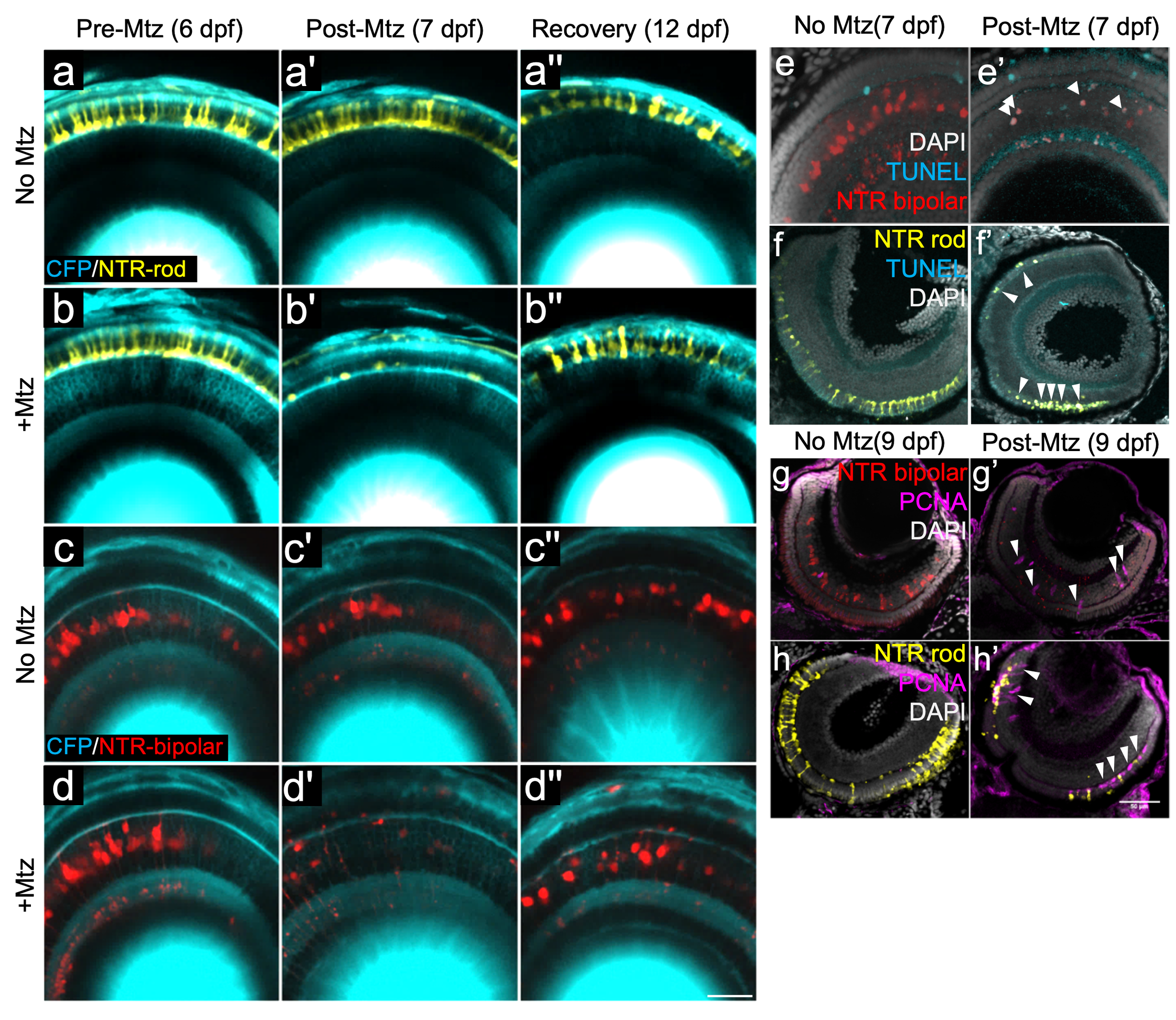

Supplement: S2 Fig — (a-b series) In vivo time-series imaging following NTR-rod larvae that were uninjured (a-a”) or received Mtz (b-b”). Images were taken in the same fish at 6 dpf (before Mtz onset in +Mtz larvae), 7 dpf (after Mtz removal) and 11 dpf (following recovery). (c-d series) In vivo time-series imaging following NTR-bipolar larvae that were uninjured (c-c”) or received Mtz (d-d”). Images were taken in the same fish at 6 dpf (before Mtz onset in +Mtz larvae), 7 dpf (after Mtz removal) and 11 dpf (following recovery). Larvae for each ablation paradigm express CFP derived from Tg(pax6-DF4:gap43-CFP)q01 to label general retinal structures (a-d series). (e-f series) Histological staining in uninjured and +Mtz eyes at 7 dpf for TUNEL in the NTR-bipolar (e-e’) and NTR-rod (f-f’) paradigms. (g-h series) Histological staining in uninjured and +Mtz eyes at 9 dpf for PCNA in the NTR-bipolar (g-g’) and NTR-rod (h-h’) paradigms. (TIF) [file pgen.1010905.s002.tif]

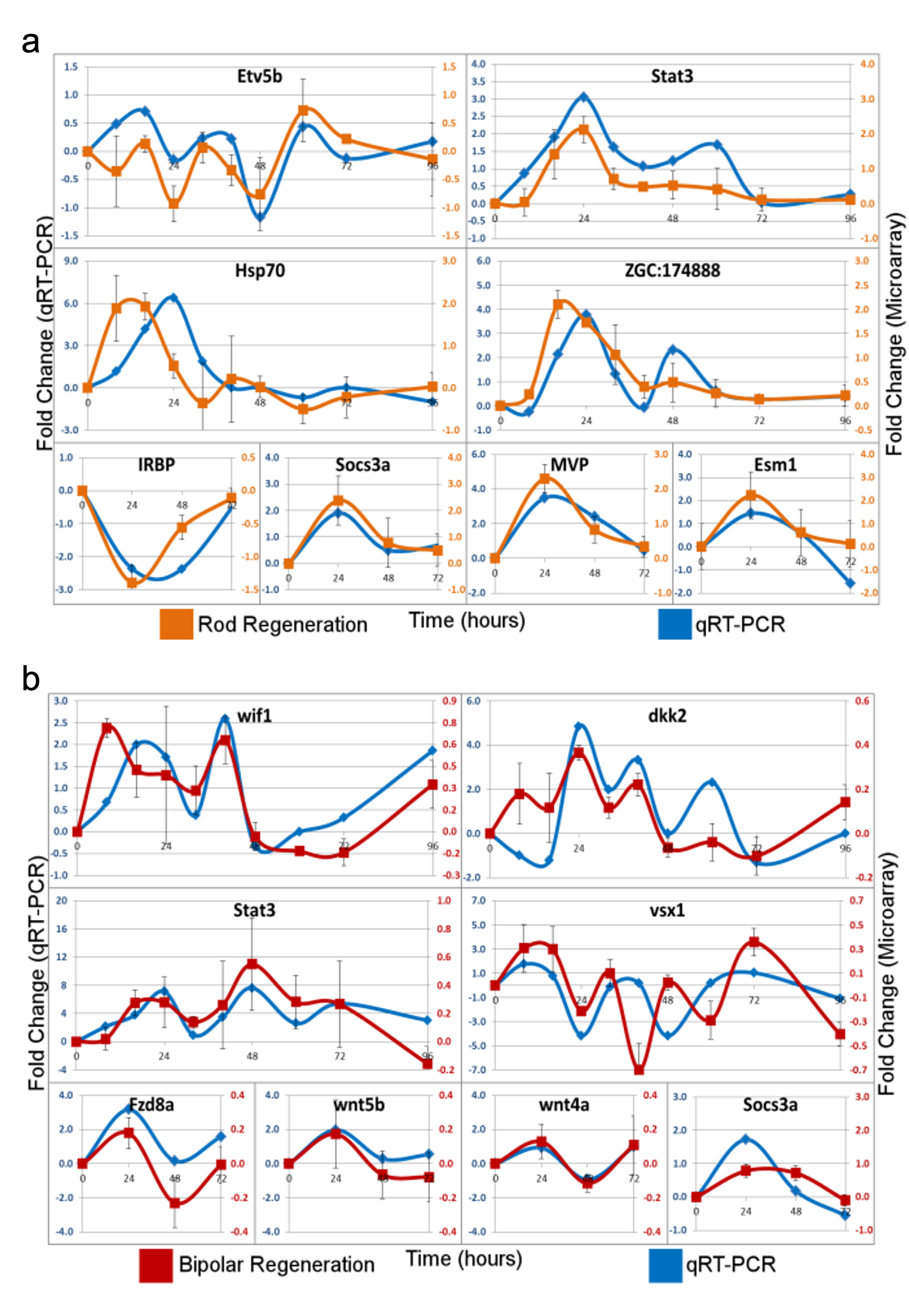

Supplement: S3 Fig — (a, b) A subset of tested genes were selected for validation using quantitative Reverse Transcription–Polymerase Chain Reaction (qRT-PCR). 8 genes were selected for a temporal analysis from 0-72h post Mtz in either the NTR-rod (A) or NTR-bipolar (B) paradigm with beta actin serving as a control. (TIF) [file pgen.1010905.s003.tif]

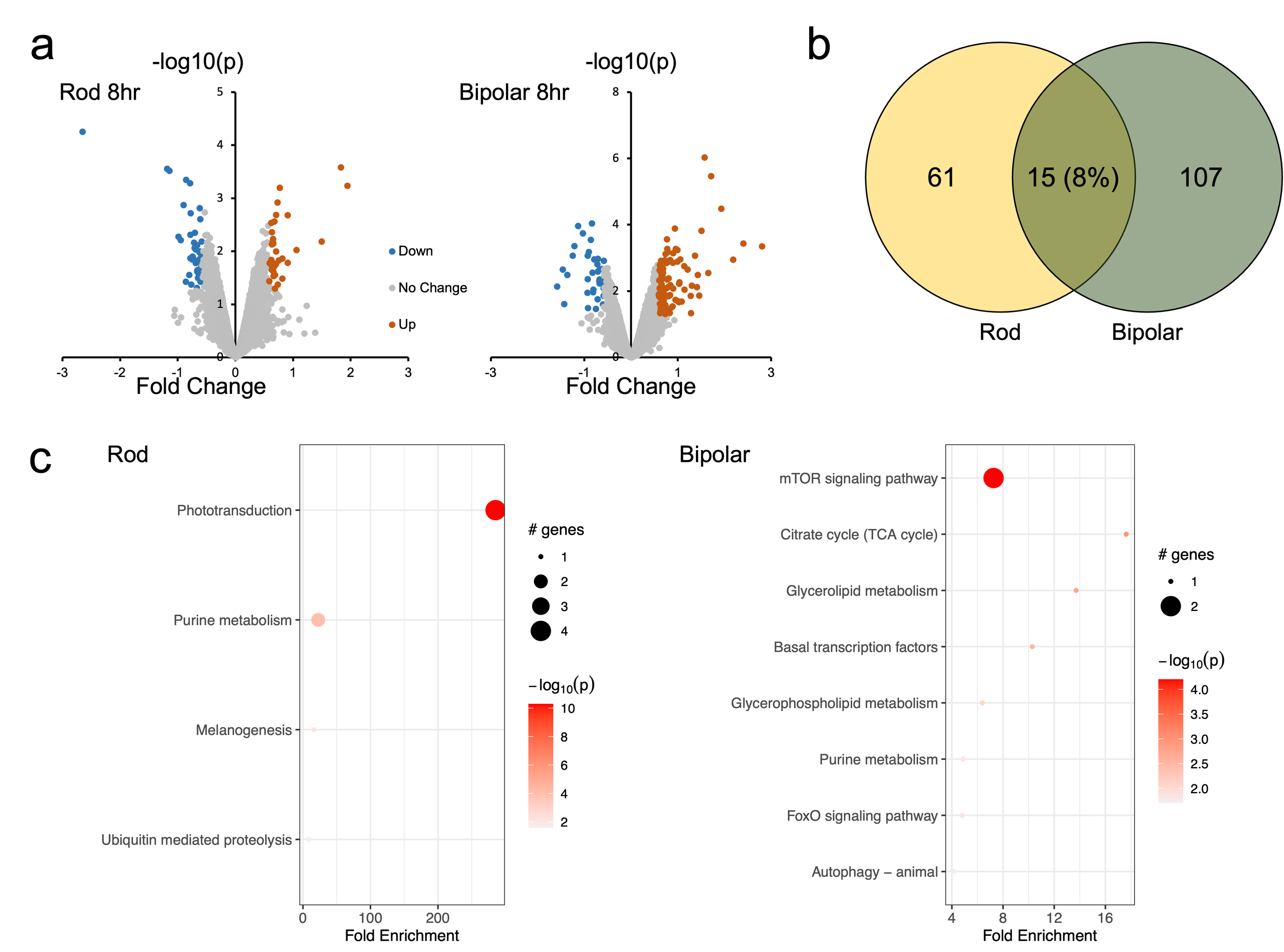

Supplement: S4 Fig — (a) Volcano plot showing the identified up (orange) and down (blue) DEGs in both NTR-rod and NTR-bipolar treatment paradigms. (b) Venn diagram showing the unique as well as shared (and % of shared) DEGs between the two paradigms. (c) KEGG pathfindR identified significantly enriched pathways in the NTR-rod and NTR-bipolar paradigms. (TIF) [file pgen.1010905.s004.tif]

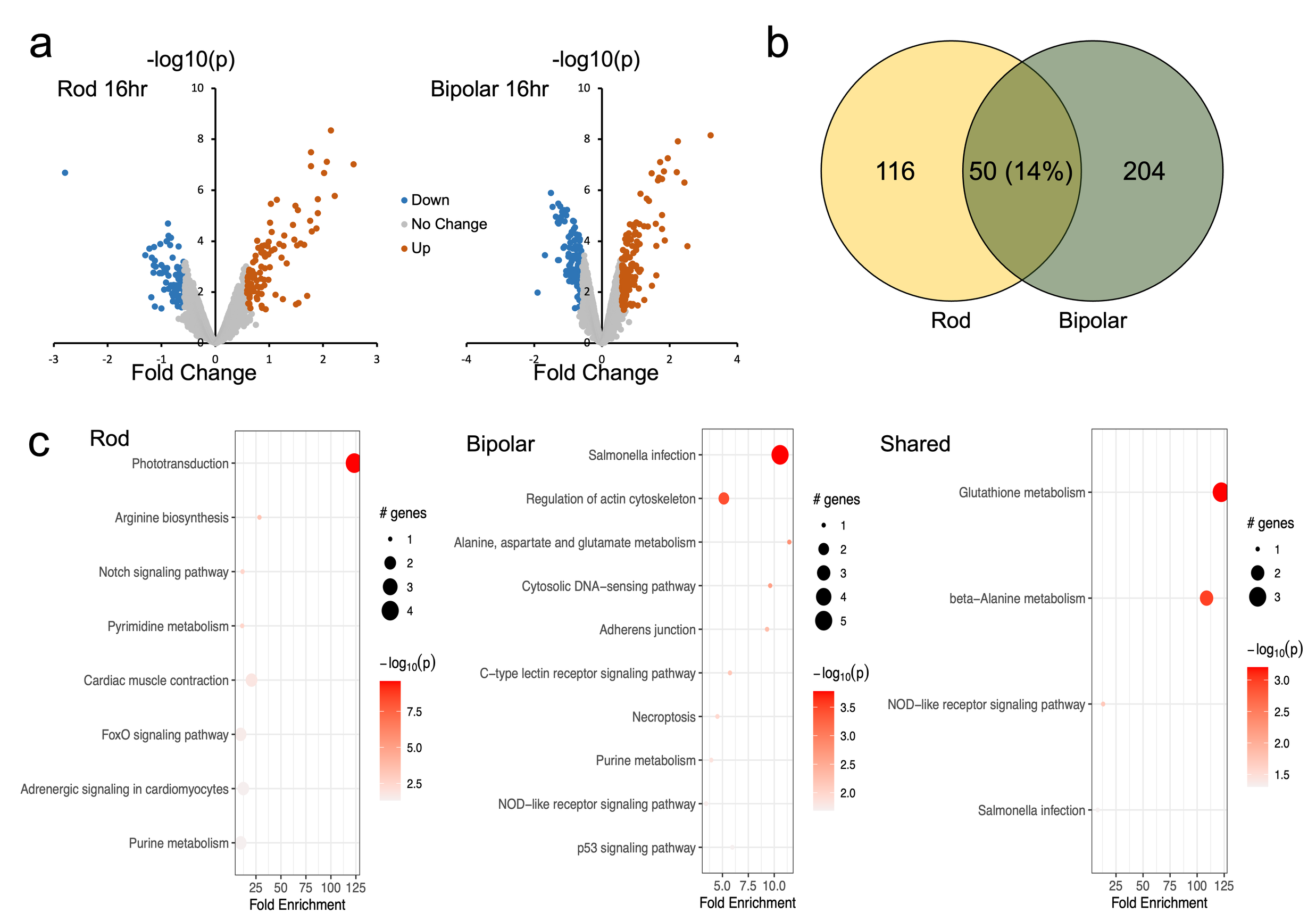

Supplement: S5 Fig — (a) Volcano plot showing the identified up (orange) and down (blue) DEGs in both NTR-rod and NTR-bipolar treatment paradigms. (b) Venn diagram showing the unique as well as shared (and % of shared) DEGs between the two paradigms. (c) KEGG pathfindR identified significantly enriched pathways in the NTR-rod and NTR-bipolar paradigms as well as those shared between the two paradigms. (TIF) [file pgen.1010905.s005.tif]

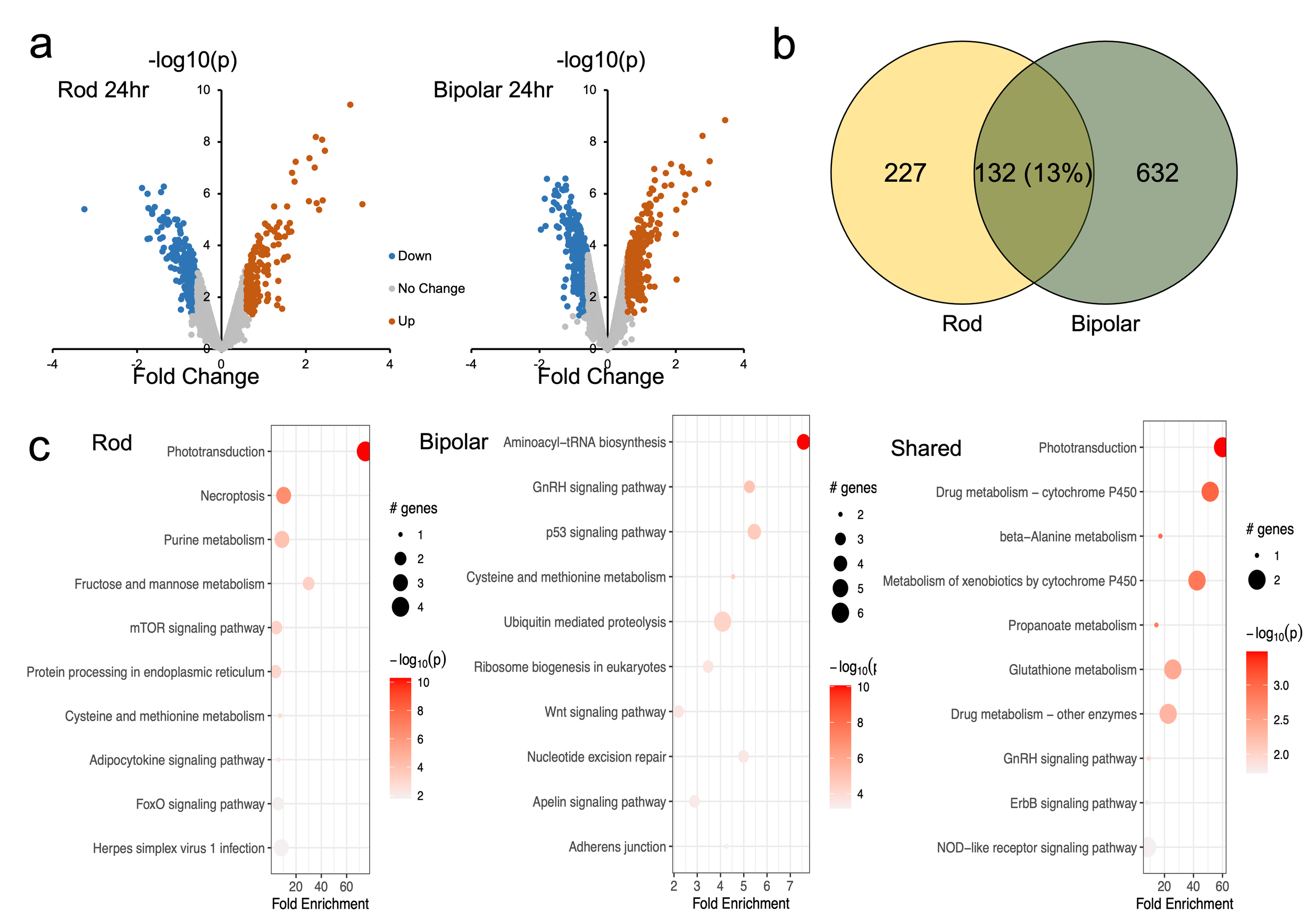

Supplement: S6 Fig — (a) Volcano plot showing the identified up (orange) and down (blue) DEGs in both NTR-rod and NTR-bipolar treatment paradigms. (b) Venn diagram showing the unique as well as shared (and % of shared) DEGs between the two paradigms. (c) KEGG pathfindR identified significantly enriched pathways in the NTR-rod and NTR-bipolar paradigms as well as those shared between the two paradigms. (TIF) [file pgen.1010905.s006.tif]

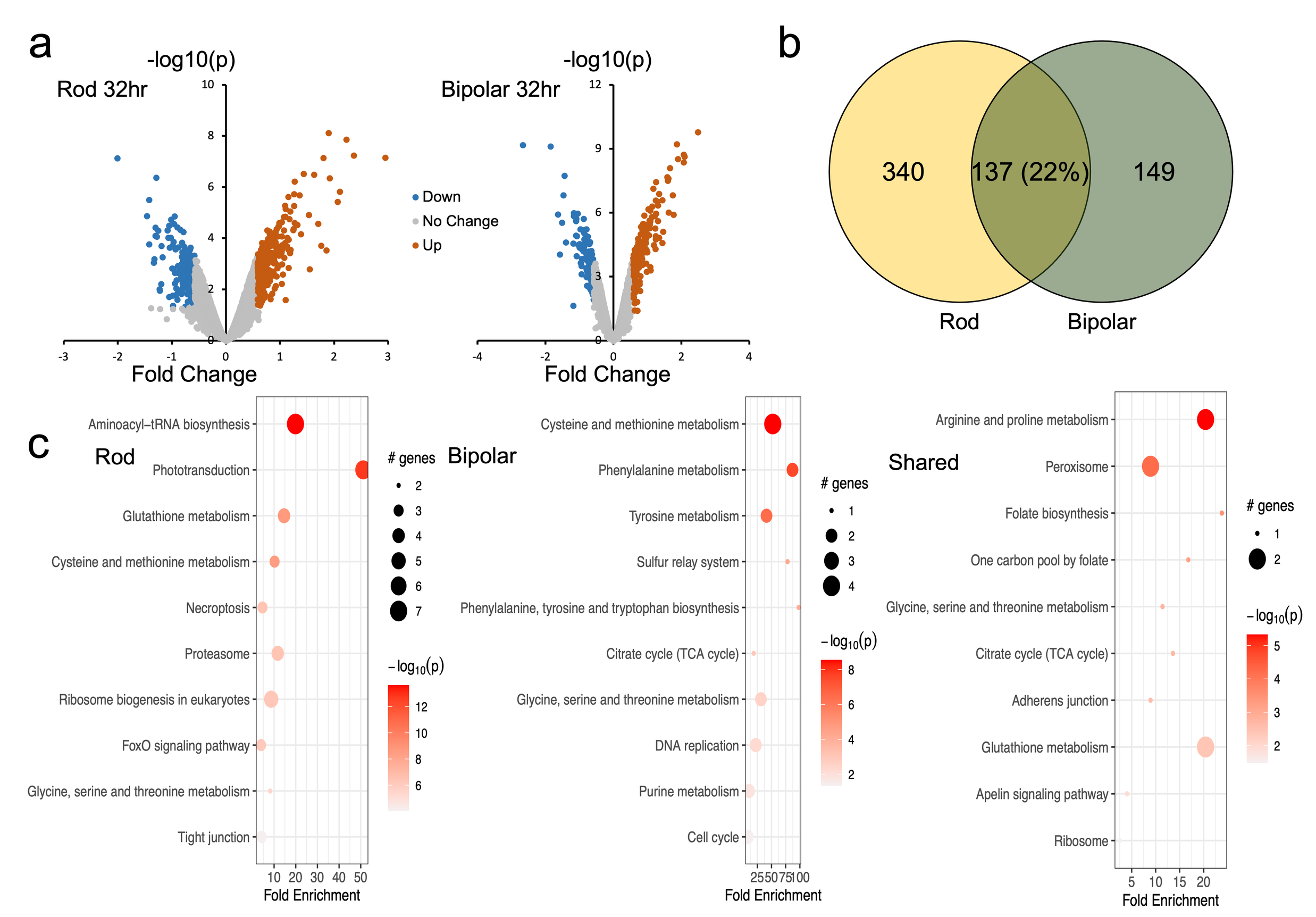

Supplement: S7 Fig — (a) Volcano plot showing the identified up (orange) and down (blue) DEGs in both NTR-rod and NTR-bipolar treatment paradigms. (b) Venn diagram showing the unique as well as shared (and % of shared) DEGs between the two paradigms. (c) KEGG pathfindR identified significantly enriched pathways in the NTR-rod and NTR-bipolar paradigms. (TIF) [file pgen.1010905.s007.tif]

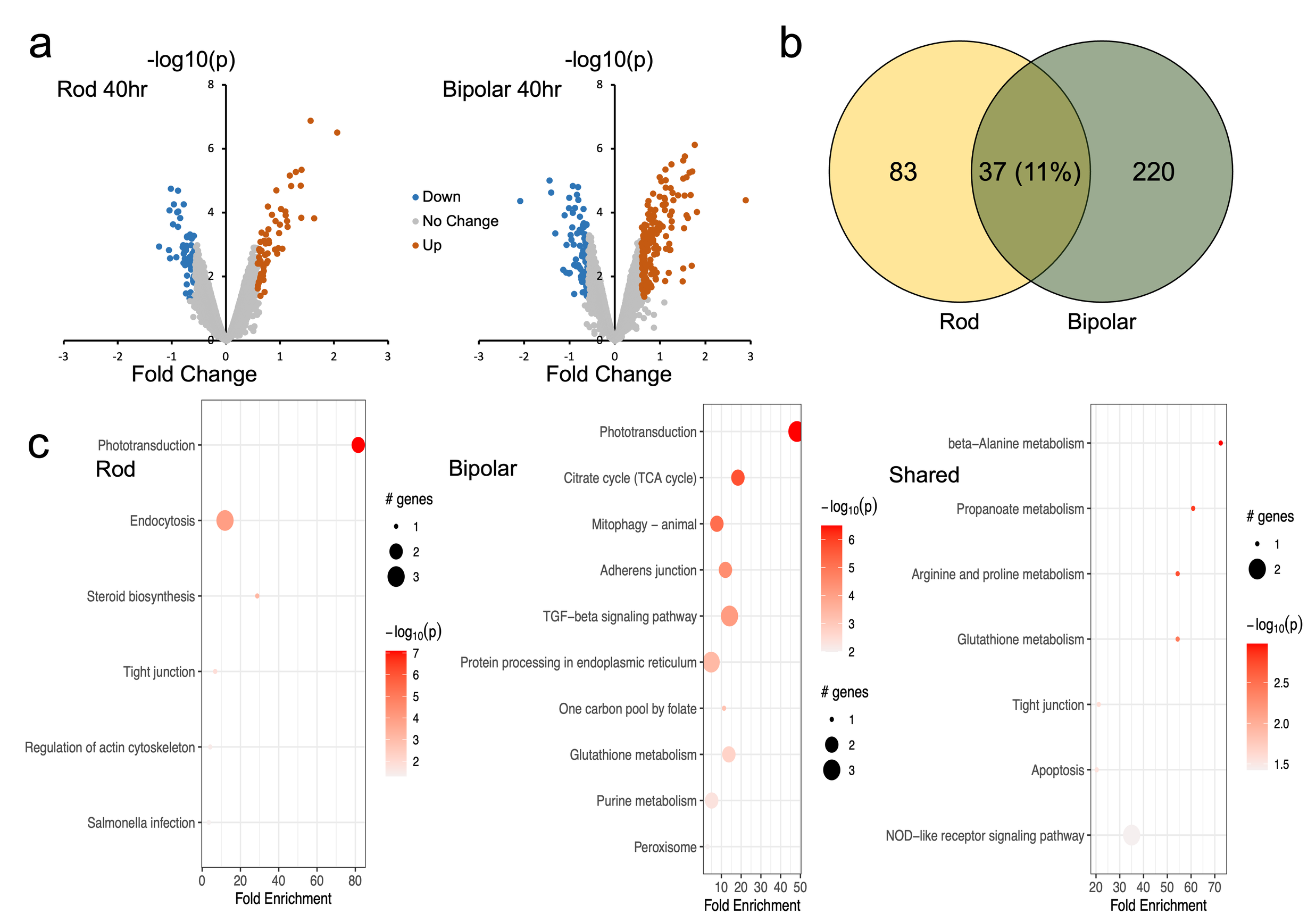

Supplement: S8 Fig — (a) Volcano plot showing the identified up (orange) and down (blue) DEGs in both NTR-rod and NTR-bipolar treatment paradigms. (b) Venn diagram showing the unique as well as shared (and % of shared) DEGs between the two paradigms. (c) KEGG pathfindR identified significantly enriched pathways in the NTR-rod and NTR-bipolar paradigms as well as those shared between the two paradigms. (TIF) [file pgen.1010905.s008.tif]

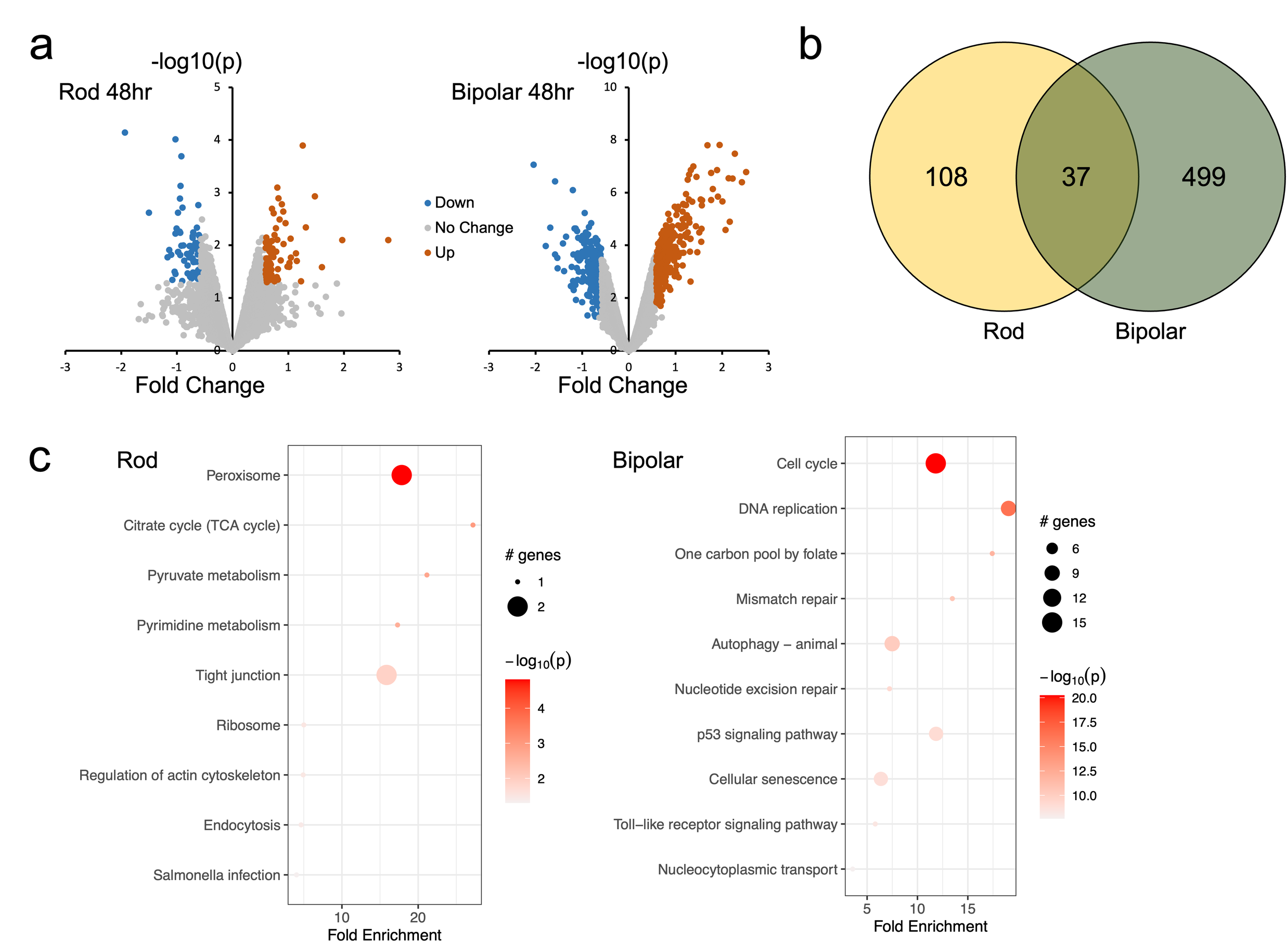

Supplement: S9 Fig — (a) Volcano plot showing the identified up (orange) and down (blue) DEGs in both NTR-rod and NTR-bipolar treatment paradigms. (b) Venn diagram showing the unique as well as shared (and % of shared) DEGs between the two paradigms. (c) KEGG pathfindR identified significantly enriched pathways in the NTR-rod and NTR-bipolar paradigms. (TIF) [file pgen.1010905.s009.tif]

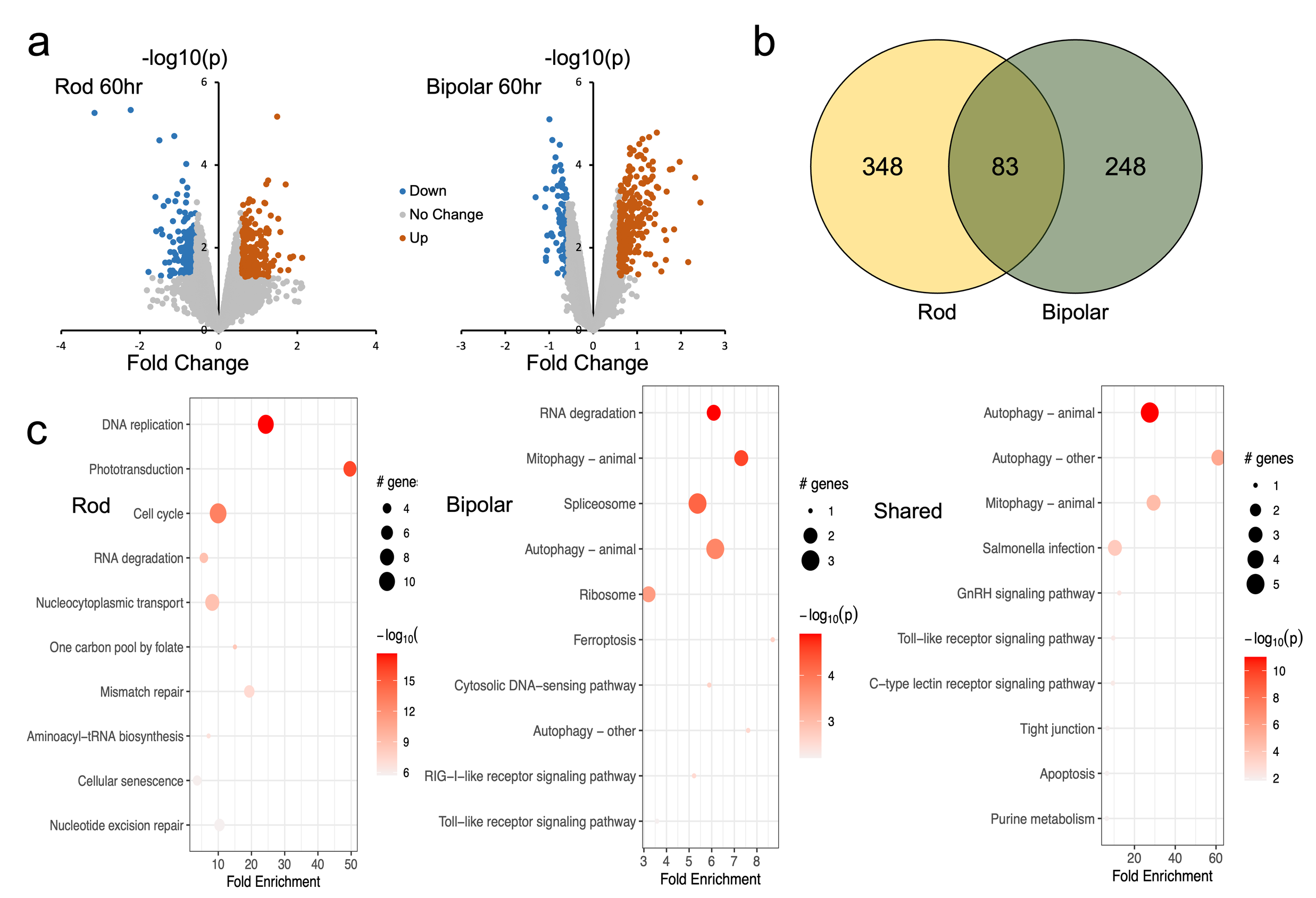

Supplement: S10 Fig — (a) Volcano plot showing the identified up (orange) and down (blue) DEGs in both NTR-rod and NTR-bipolar treatment paradigms. (b) Venn diagram showing the unique as well as shared (and % of shared) DEGs between the two paradigms. (c) KEGG pathfindR identified significantly enriched pathways in the NTR-rod and NTR-bipolar paradigms as well as those shared between the two paradigms. (TIF) [file pgen.1010905.s010.tif]

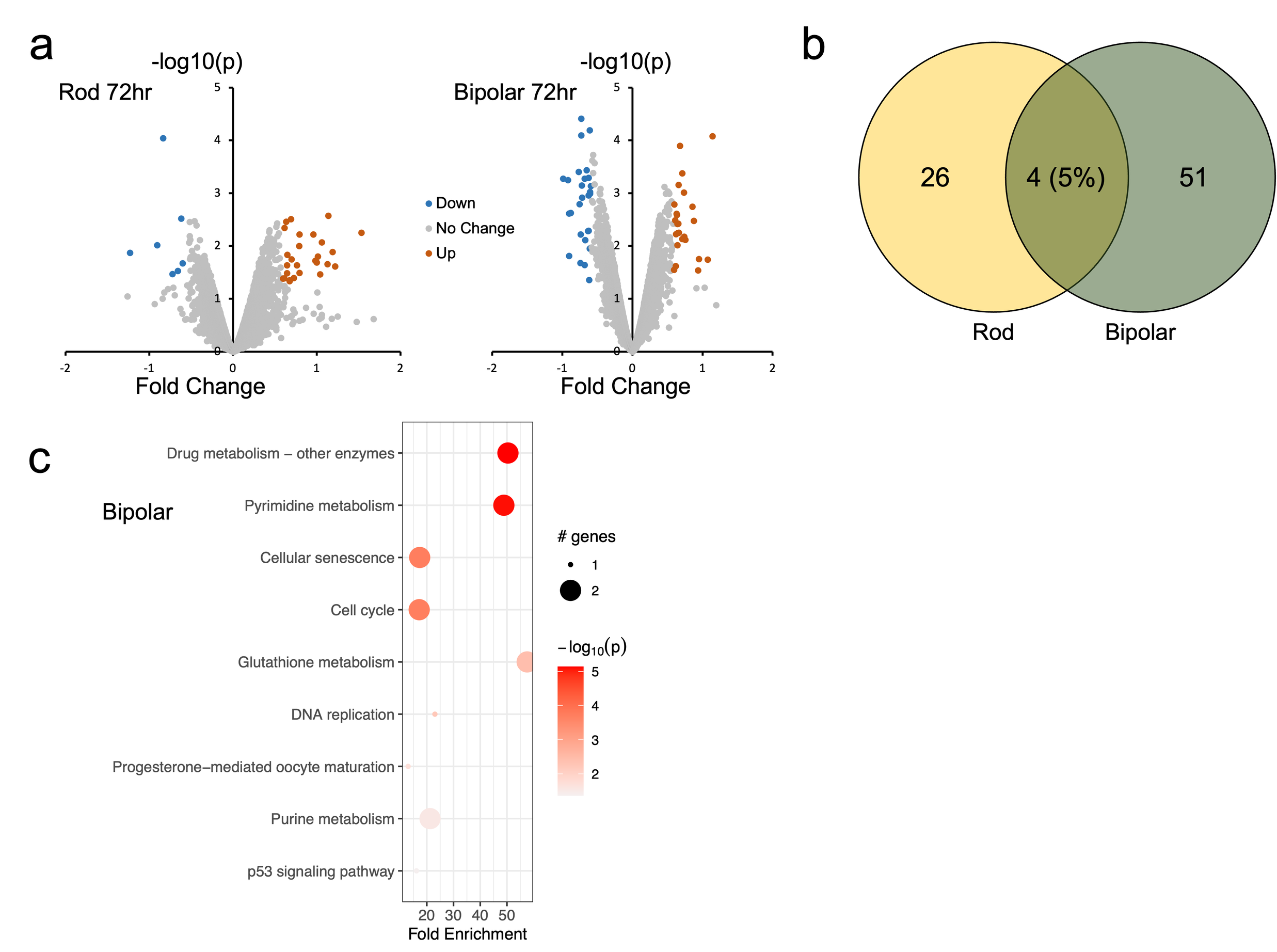

Supplement: S11 Fig — (a) Volcano plot showing the identified up (orange) and down (blue) DEGs in both NTR-rod and NTR-bipolar treatment paradigms. (b) Venn diagram showing the unique as well as shared (and % of shared) DEGs between the two paradigms. (c) KEGG pathfindR identified significantly enriched pathways in the NTR-bipolar paradigm. (TIF) [file pgen.1010905.s011.tif]

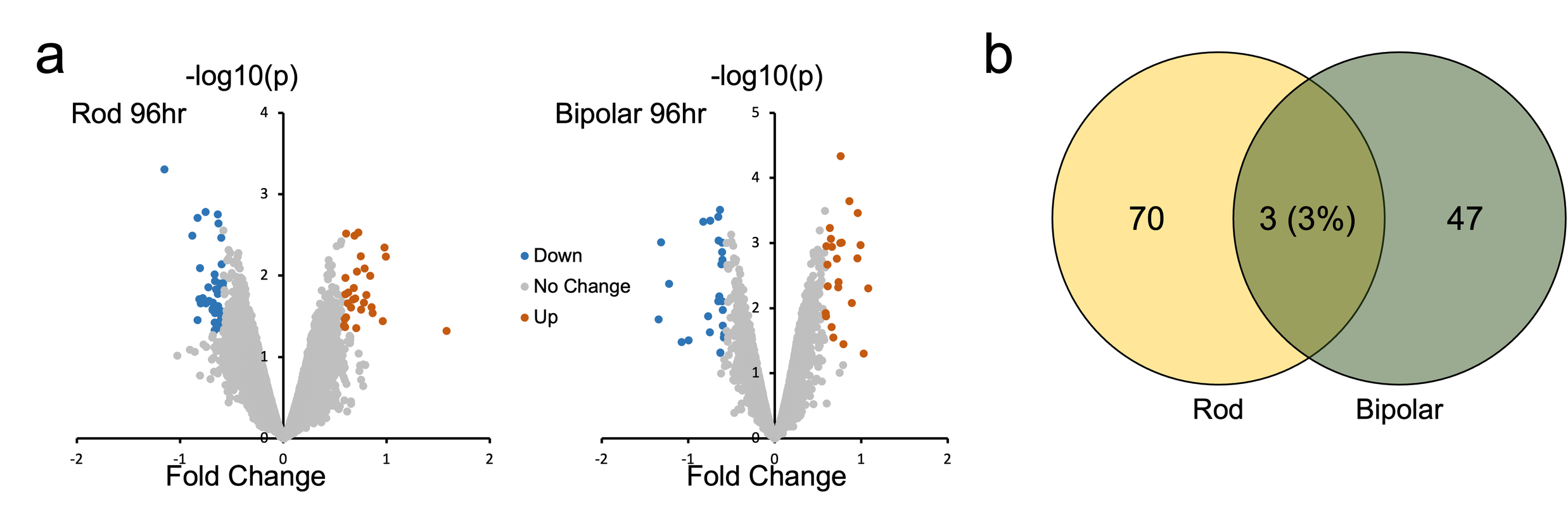

Supplement: S12 Fig — (a) Volcano plot showing the identified up (orange) and down (blue) DEGs in both NTR-rod and NTR-bipolar treatment paradigms. (b) Venn diagram showing the unique as well as shared (and % of shared) DEGs between the two paradigms. (TIF) [file pgen.1010905.s012.tif]

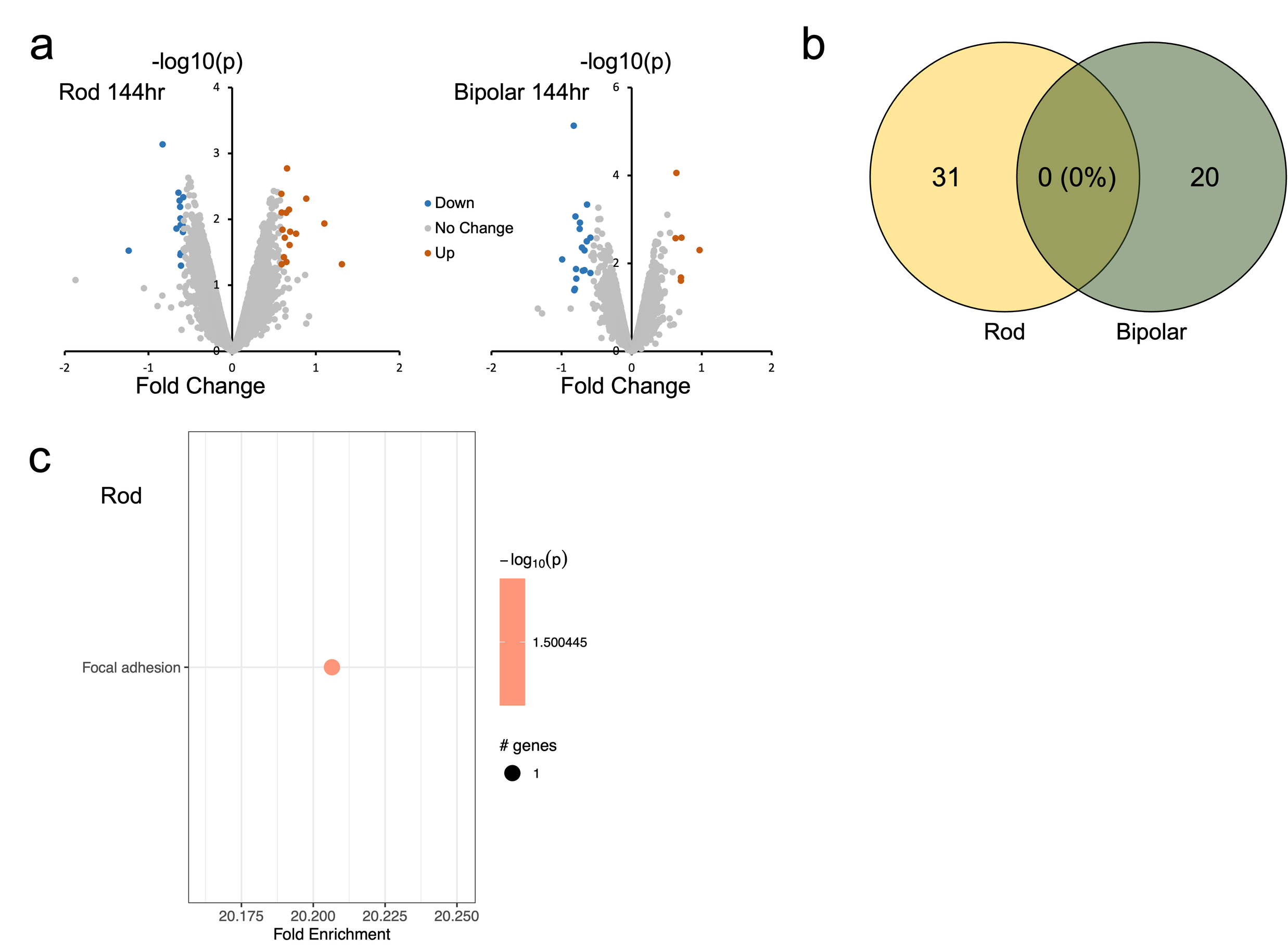

Supplement: S13 Fig — (a) Volcano plot showing the identified up (orange) and down (blue) DEGs in both NTR-rod and NTR-bipolar treatment paradigms. (b) Venn diagram showing the unique as well as shared (and % of shared) DEGs between the two paradigms. (c) KEGG pathfindR identified significantly enriched pathways in the NTR-rod paradigm. (TIF) [file pgen.1010905.s013.tif]

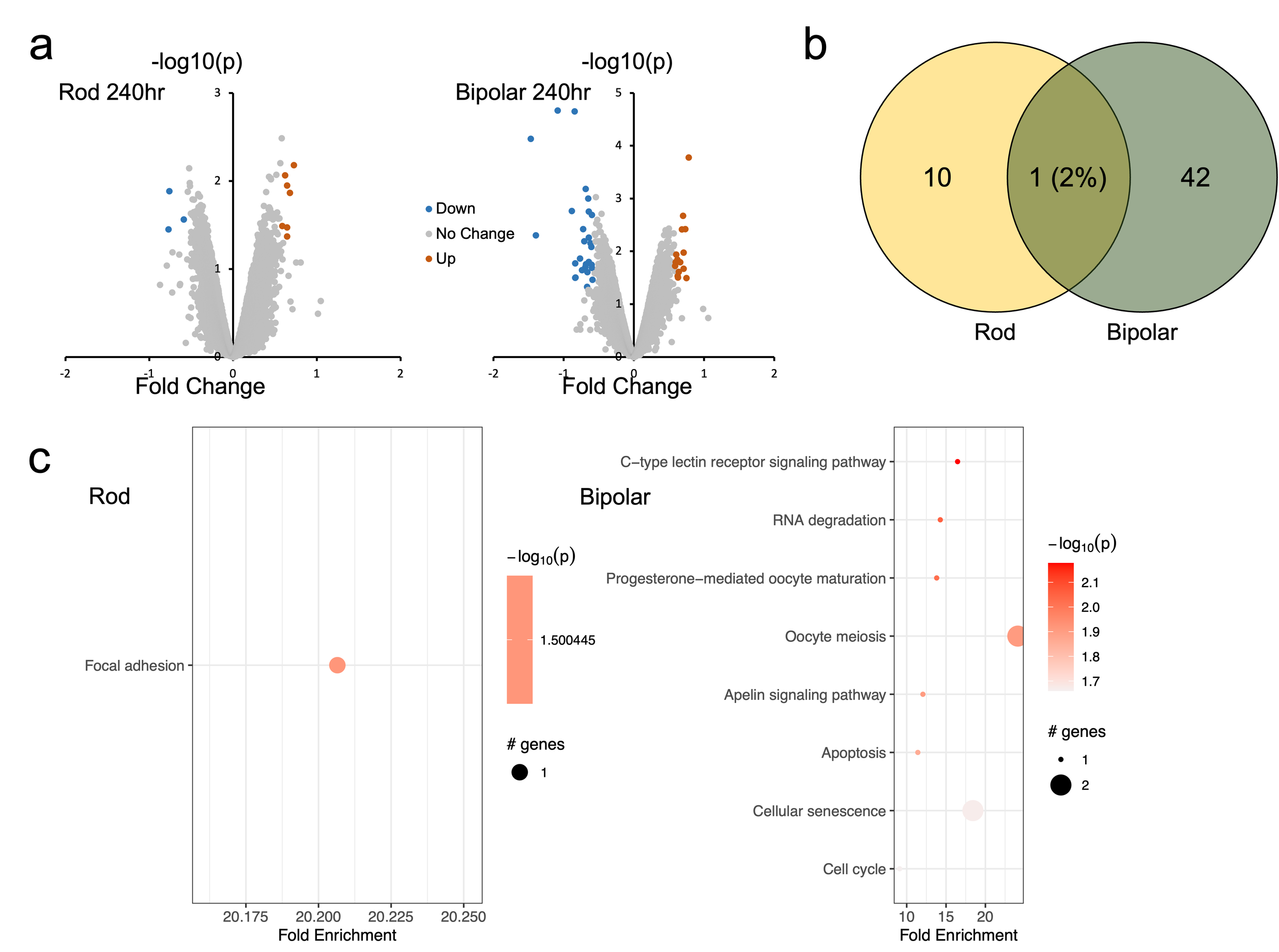

Supplement: S14 Fig — (a) Volcano plot showing the identified up (orange) and down (blue) DEGs in both NTR-rod and NTR-bipolar treatment paradigms. (b) Venn diagram showing the unique as well as shared (and % of shared) DEGs between the two paradigms. (c) KEGG pathfindR identified significantly enriched pathways in the NTR-rod and NTR-bipolar paradigms. (TIF) [file pgen.1010905.s014.tif]

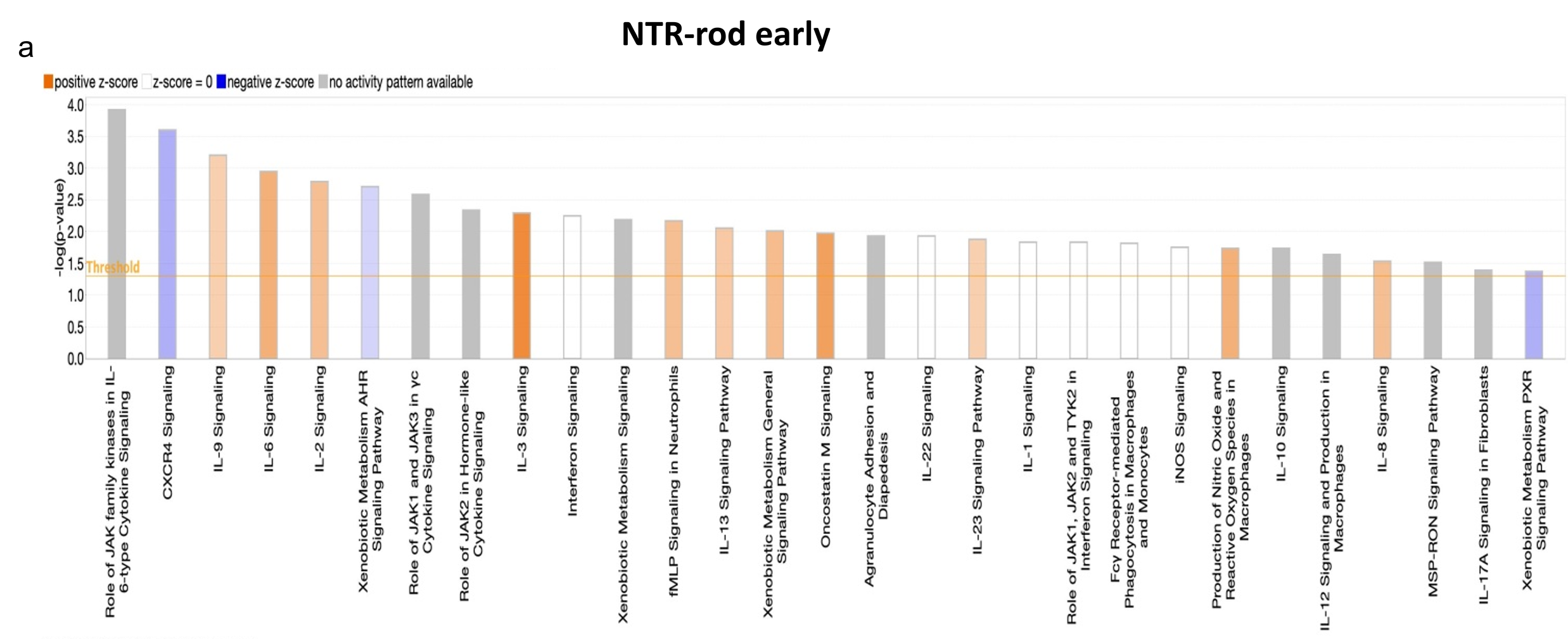

Supplement: S15 Fig — (a-d) IPA was used to test for enriched immune related pathway terms from DEGs for the NTR-rod early pattern. (TIF) [file pgen.1010905.s015.tif]

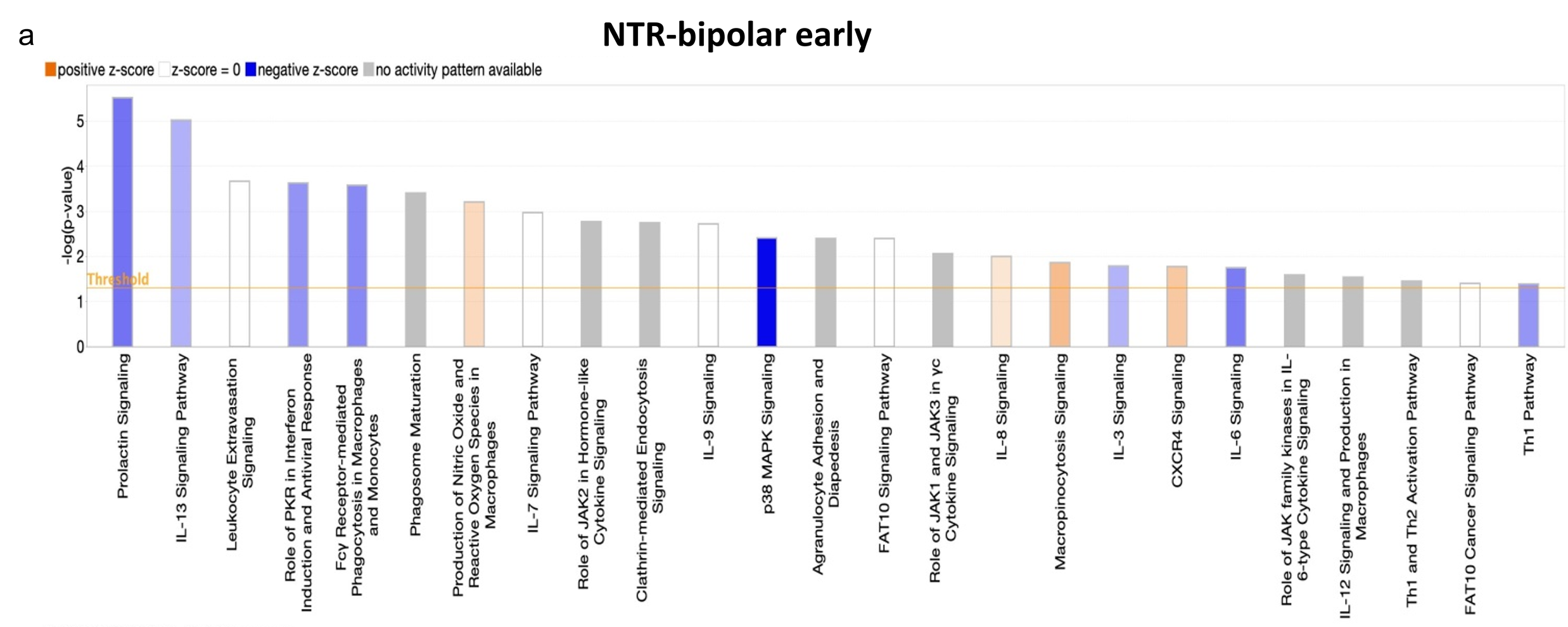

Supplement: S16 Fig — (a-d) IPA was used to test for enriched immune related pathway terms from DEGs for the NTR-bipolar early pattern. (TIF) [file pgen.1010905.s016.tif]

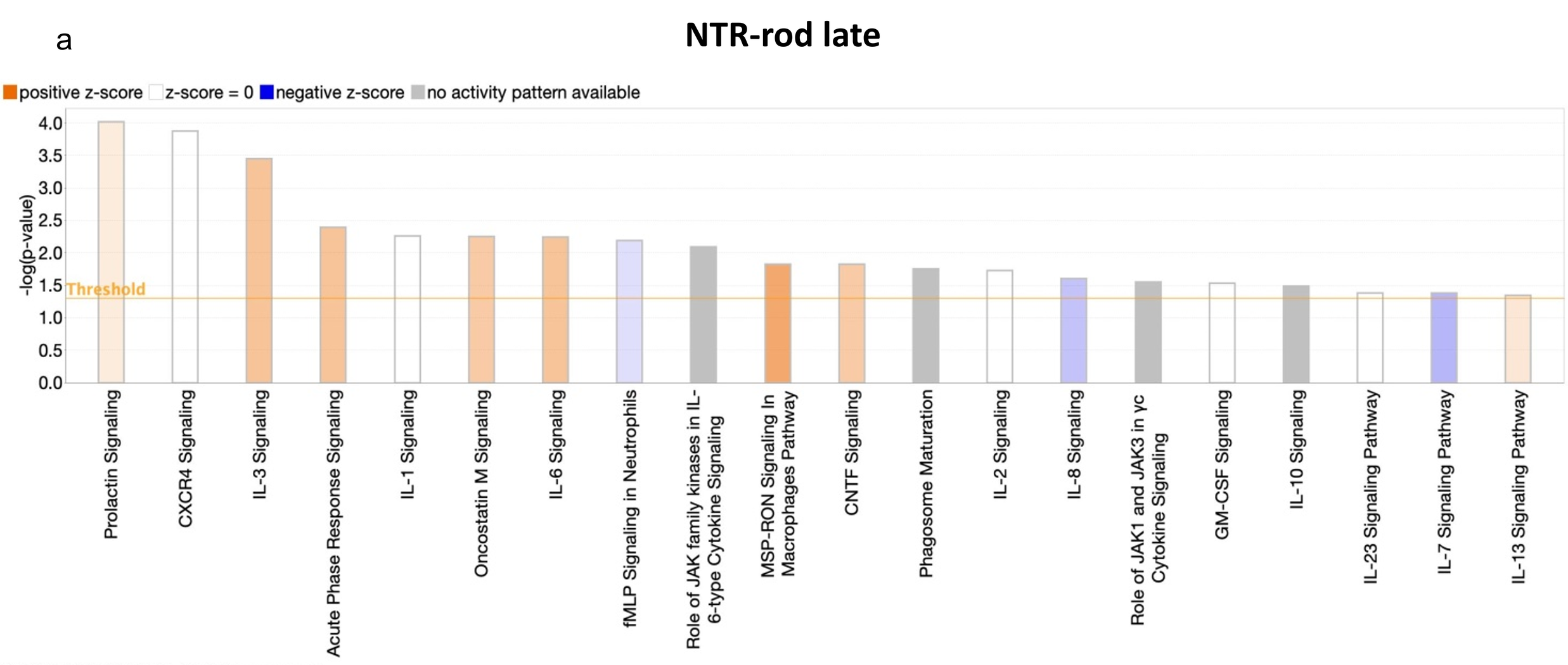

Supplement: S17 Fig — (a-d) IPA was used to test for enriched immune related pathway terms from DEGs for the NTR-rod late pattern. (TIF) [file pgen.1010905.s017.tif]

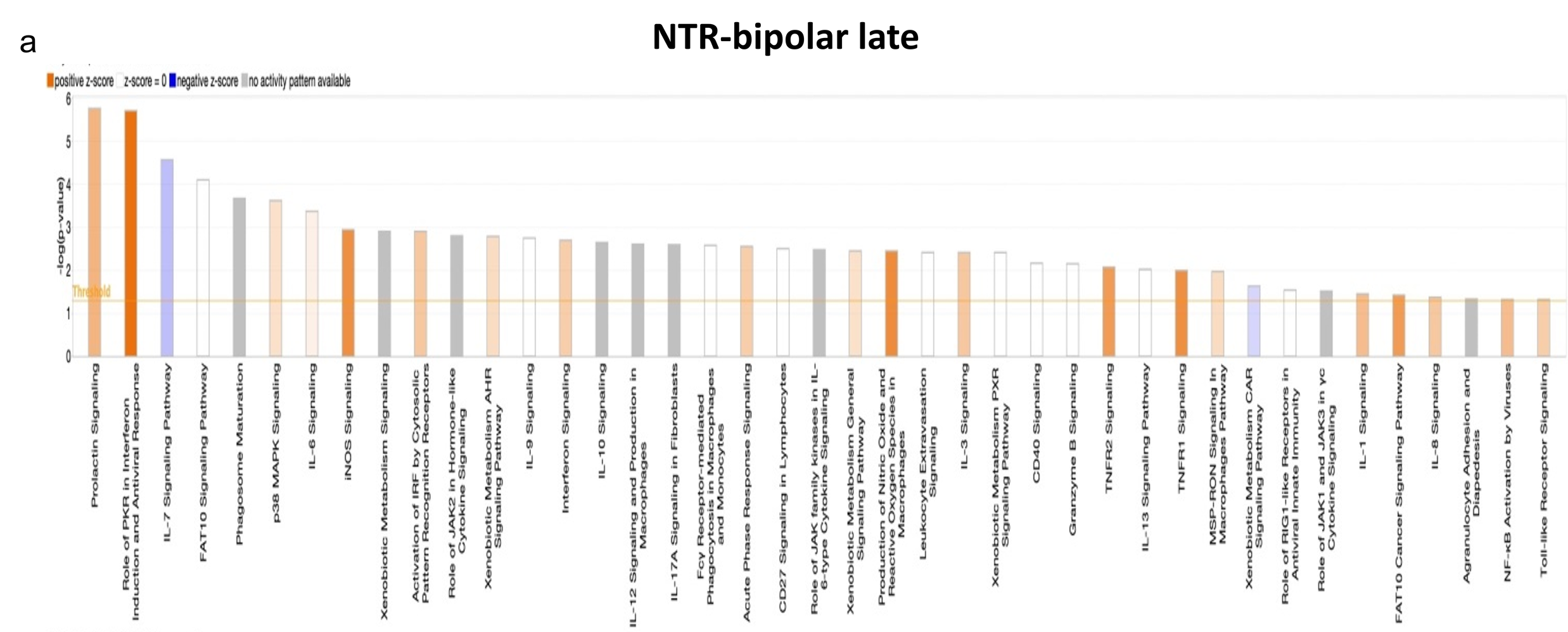

Supplement: S18 Fig — (a-d) IPA was used to test for enriched immune related pathway terms from DEGs for the NTR-bipolar late pattern. (TIF) [file pgen.1010905.s018.tif]

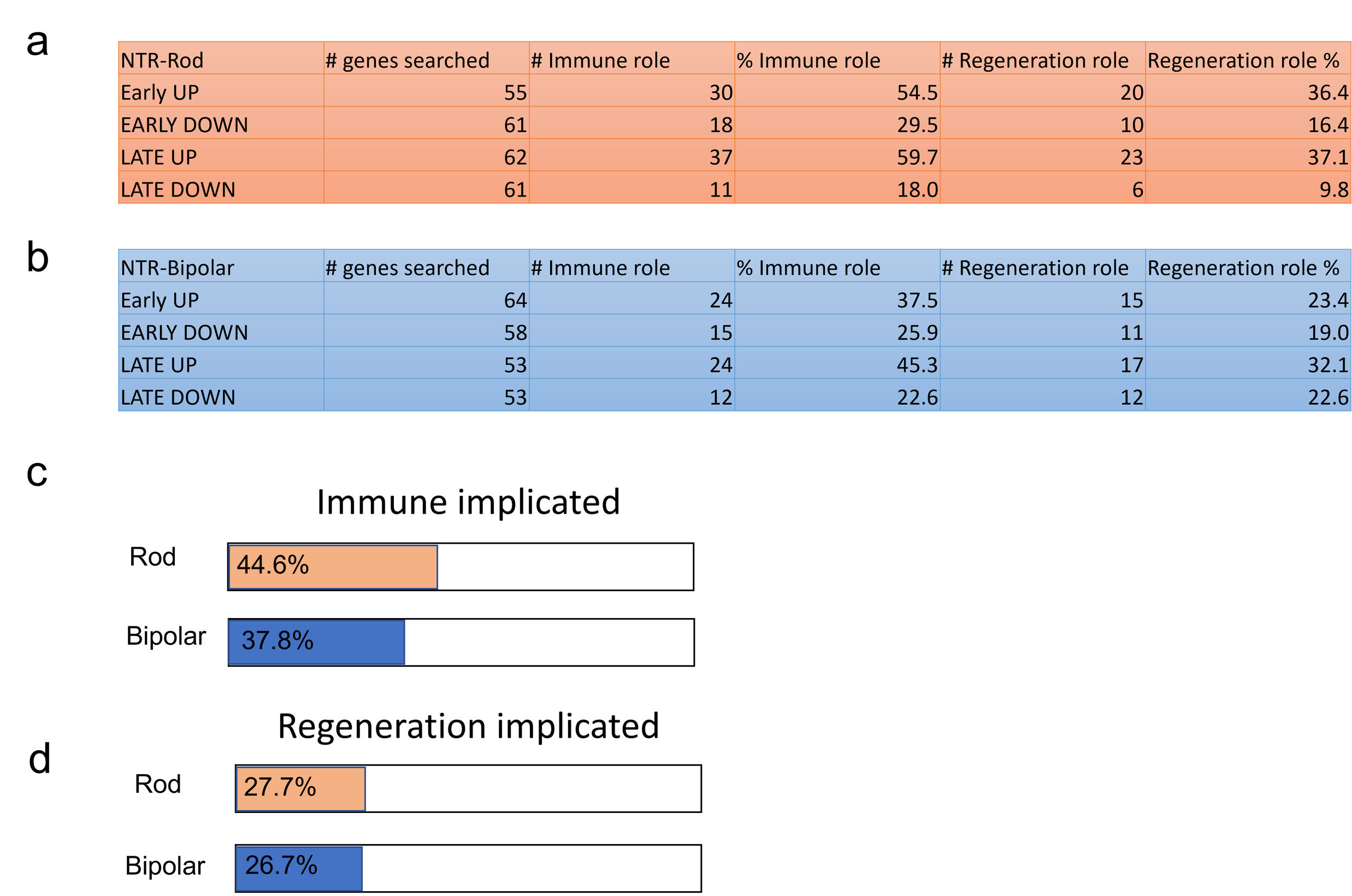

Supplement: S19 Fig — (a-b) The top ~50–60 DEGs for the NTR-rod (a) and NTR-bipolar (b) patterns were searched in the literature for any links to a role in the immune system or in regeneration. The percentage of those with a link to each term are shown. (c-d) Bar showing the percentage of top DEGs that were implicated in the immune system (c) or regeneration (d). (TIF) [file pgen.1010905.s019.tif]

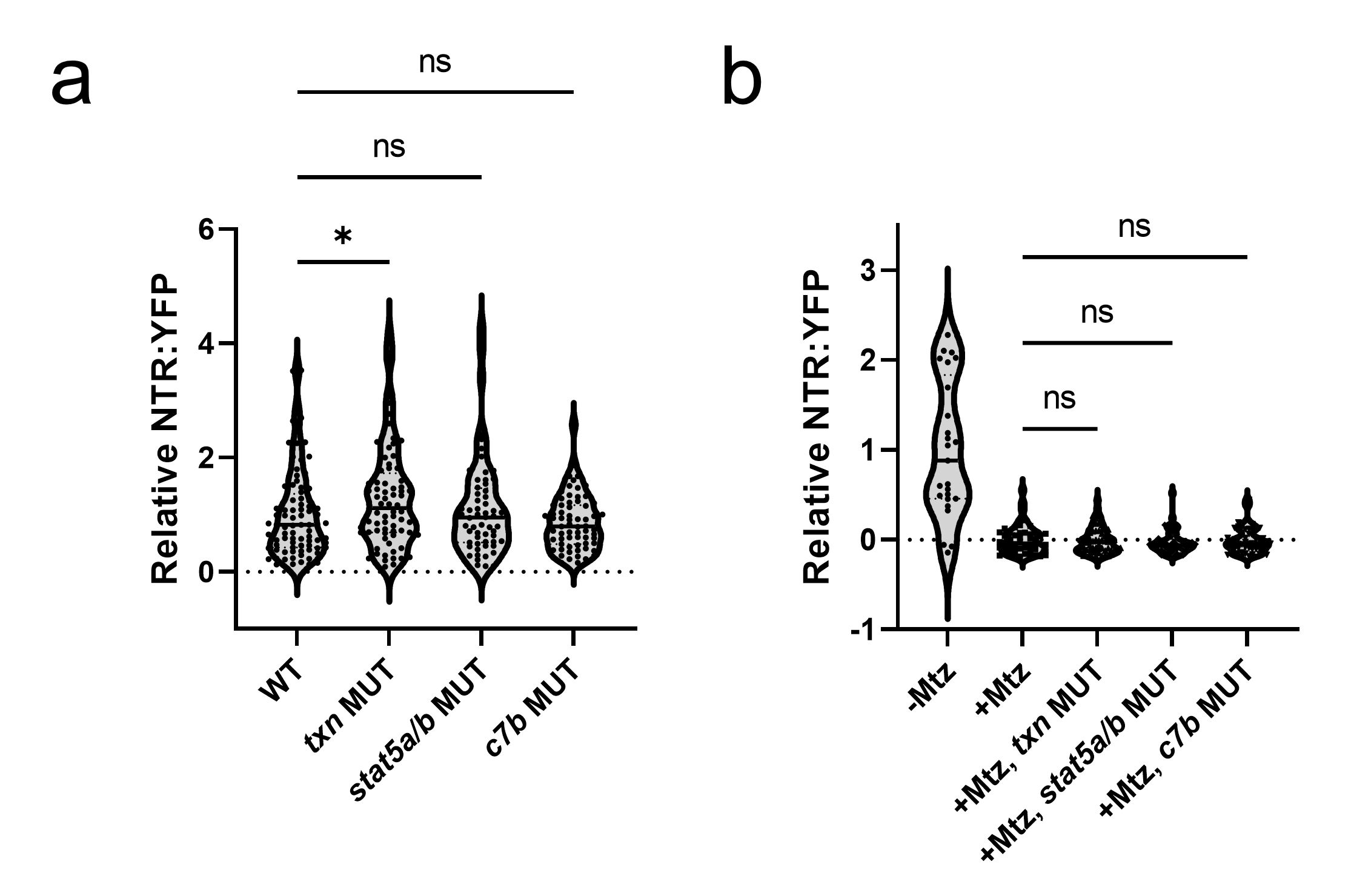

Supplement: S20 Fig — (a) Quantification of rod cell development pre-ablation by plate reader assay at 5 dpf in wildtype (WT) and MUT larvae for target genes txn, stat5a/b and c7b. (b) Quantification of rod cell ablation following Mtz treatment by plate reader assay at 7 dpf in non-ablated (-Mtz) and ablated controls (+Mtz) and ablated MUT larvae for target genes txn, stat5a/b and c7b. For statistical comparisons, Welch’s one-way ANOVA was followed by student’s t test with Dunnett’s method for multiple comparisons correction. Asterisks indicate the following p-value ranges: * = p<0.05, ** = p<0.01, *** = p<0.001, and **** = p<0.0001, “ns” indicates p>0.05. (TIF) [file pgen.1010905.s020.tif]
