# Supplementary material for: Transcriptomic comparison of two selective retinal cell ablation paradigms in zebrafish reveals shared and cell-specific regenerative responses
Source: PLoS Genet. 2023 Oct 11;19(10):e1010905. doi: 10.1371/journal.pgen.1010905 (PMC10593236; doi:10.1371/journal.pgen.1010905)
Supplement: Attachment — Submitted filename: 20230621_PlosGeneticsReviewerResponse_ToSUBMIT.docx [file pgen.1010905.s029.docx]

Reviewer's Responses to Questions

**Comments to the Authors:**
 **Please note here if the review is uploaded as an attachment.**

Reviewer #1: In the article by Emmerich, et al., the Authors leverage prior technologies created in the laboratory of Dr. Mumm to investigate transcriptomic changes across the regeneration of the zebrafish retina in two different, cell type-specific ablation paradigms. This work assesses the functional consequence of loss of individual cell fates, serving as a proxy to degenerations that affect the human retina and providing insights into the gene regulatory networks that promote cell-type specific regeneration in zebrafish and/or limit the regenerative ability of the human retina.

The manuscript does a good job of providing details of background and rationale for the studies, technologies utilized, interpretations of transcriptomic and pathway analyses, and providing context of the discoveries within prior research.

Additionally, the Authors provide biological relevance for findings of stat3 differential expression.

Areas to be addressed:

Minor Points:
 1) Fig. 1 - l and m - please label the figure with the treatment paradigm that each panel represents

Author Response: Figure labels have been added to indicate the treatment paradigm for l and m (NTR:rod and NTR:bipolar, respectively).

2) Statistics:
 - All figures with statistical analyses: please provide the statistical test utilized for each analysis within the figure legend where statistical data is presented. It is unclear which panels utilized t-tests versus One-way ANOVAs based on the presented data/methods

- no statistics on figure 6 c

- line 230-233 - the authors make comparisons between differences in location of proliferative cells between rod and bipolar ablation paradigms. However, no such statistical comparison is actually presented.

- Appropriateness of the statistical methods utilized. Please provide rationale for the use One-Way ANOVA (Figure 1l-m; Figure 6 i). In these analyses, the effect of 2 different variables is being tested; either Region (rod precursor or Muller glia proliferation) and MTZ treatment (Figure 1); or Region (rod precursor or Muller glia proliferation) and stat3 expression (Figure 6). It seems like a two-way ANOVA would be more appropriate.

Author Response:

*Statistical analyses*: We now indicate in the appropriate figure legends the statistical tests used. The following was also added to the Methods for additional clarity, “For statistical comparisons, Student's t test was used to assess paired conditions while Welch's one-way ANOVA followed by Dunnett's test for multiple comparisons was used to assess differences across more than two conditions.” Lines 700-703.

*No statistics*: Previously, statistically non-significant differences were not indicated graphically (e.g. 6c). We now show the results of all comparisons calculated no matter the level of statistical significance. We have added “ns” for all comparisons showing non-significant statistical differences.

*Comparisons between rod and bipolar ablation paradigm*: To bolster the value of our cross-paradigm comparison, we feel it is important to stress that the proliferative response of INL-localized MG/MGPC cells is similar between the rod and bipolar ablation paradigms. Accordingly, we have added means ±sd and p-values for these comparisons within the text at lines 226-232. Student's t test was used to calculate p-values between the paired conditions.

*Appropriate statistics*: We have updated our discussion of the statistics to correct our error. For most aspects of the study, we analyzed the effects of a single variable (Mtz) and/or compared only two conditions. Accordingly, Student's t test was used to assess differences between paired conditions. To address potential confusion due to our inclusion of multiple paired tests in a single graph, we have reformatted and colorized graphs to visually indicate what the paired comparisons were (Fig. 1l, m and Fig. 6i).

Where more than two conditions were tested/compared (Fig. 1g and 7b), Welch's one-way ANOVA was followed by student's t test with Dunnett’s method for multiple comparisons correction. As we did not test two independent variables, in combination, on a dependent variable (e.g., proliferation), we feel a one-way ANOVA is the appropriate test.

3) KEGG pathway analyses would be more informative if data was presented with up- and down-regulated genes separated. For example, in Supp. Fig 4C - KEGG pathway analysis of ALL differential genes shows an 'enrichment' of phototransduction genes. It would be more informative if all down-regulated genes were analyzed indepedently of 'up-regulated' genes and 'phototransduction' was significantly enriched, consistent with loss of rod photoreceptors. Analyzing both up and down-regulated genes together may actually be reducing the significance of pathways that are affected.

Author Response: We appreciate this view. Initially, our strategy was indeed to keep up/down DEG lists separate for pathway analyses. However, we found more power in analyzing the data as a combined set - finding more significantly modulated pathways. This likely reflects that within a given network some genes are upregulated while others are downregulated to activate signaling – i.e., that networks involve feedback circuits with complex genetic interactions. Given that this approach enriched our results, we elected to present the data from this perspective. However, by providing the full list of DEGs, readers will be able to easily perform any alternative pathway analyses of interest.

4) Data availability - Unclear if the Microarray data has been submitted to a publicly available resources (GEO)

Author Response: The microarray data is now available at GEO at accession number GSE234646, and the paper now indicates this in the methods section under Data Availability (Lines 753-755).

Major Point:
The goal of the study is to provide evidence of differential regenerative responses at the transcriptomic level based on specific cell-type ablation paradigms. This goal is met through the comprehensive microarray analyses. However the biological relevance of these findings remains limited.

*stat3* has already been implicated in regulating regenerative responses in both fish and mice, a finding acknowledged by the Authors with appropriate references. To achieve a goal of identifying cell type-specific ablation responses, the authors would provide one or both of the following:

1) Proliferative response in bipolar ablation paradigm with/without loss of stat3; direct comparisons to rod ablation response. If stat3 is most important for Muller glia-derived regeneration, one may anticipate a more drastic inhibition of bipolar cell regeneration. An observation of no-difference compared to rod-ablation would suggest that stat3 is required for both rod precursor and Muller glia-derived regeneration, supporting the transcriptomic data where stat3-signaling was affected in both paradigms

2) Examination of the regenerative responses when a bipolar-cell ablation 'hub' gene is reduced (PPARG), in both the rod and bipolar ablation paradigms. The differential activity of the KEGG pathway analyses might suggest differential response.

It should be noted that any result from either/both of these suggested experiments would be informative.

Author Response: We appreciate these suggestions and agree that increasing the biological relevance of our observations would enrich the study. Accordingly, we have now tested roles for a total of 7 genes in rod cell regeneration, including factors implicated solely in the bipolar ablation paradigm (*stat5a/b* and *pparg*, new Fig. 7).

As the reviewer suggests, we also wanted to carry out equivalent functional gene analyses in the bipolar regeneration paradigm. Unfortunately the bipolar line, which employs the Gal4/UAS system, has undergone epigenetic silencing of the UAS-linked NTR-Cherry transgene – a known issue for these resources (see studies from Mary Goll and colleagues). Progressive reduction of expression of the UAS:NTR-Cherry transgene has rendered us unable to (1) quantify bipolar cell regeneration using our plate reader assay, and (2) to be certain that bipolar cell ablation levels are equivalent to the original assays used to derive DEGs (which occurred several years ago). During these revisions the extension we requested was to explore alternative options for overcoming these obstacles but unfortunately, to date, these efforts have not been successful.

We are still actively working on potential methods to revive this line for future experiments, including: (1) using new CRISPR/Cas9 tools to direct demethylase proteins to UAS sites to reverse silencing, and (2) develop a new driver/reporter line using our newly published NTR2.0 reporters (that enable more efficient ablation) along with a new driver to better direct expression to bipolar cells (using single cell genomics datasets to identify the best marker genes).

Despite our inability to perform crispant tests with the bipolar line, we feel the added tests in the rod line meet the overarching goal of this comment to improve biological relevance. The new figure 7 shows the effects of mutating 6 new genes on rod regeneration: mutation of *pparg,* a gene implicated solely in the bipolar paradigm*,* had no effect on rod regeneration, and mutation of 3 new genes significantly enhanced rod regeneration (*stat5a/b*, *c7b*, and *txn*). Importantly, all of the hit genes are immune-related factors, tying these results to our DEG results which strongly implicate immune signaling in both paradigms. As a side note, we are also testing many of these same genes in a new retinal ganglion cell (RGC) ablation paradigm. Consistent with the reviewers point, each tested gene has shown differential effects on RGC versus rod regeneration. We anticipate publishing this data in the near future. We hope that this slight delay in the comparative aspect of our crispant studies will be acceptable and that the new test add sufficient biological relevance to warrant publication.

Reviewer #2: Kevin Emmerich and colleagues use microarray transcriptomics to characterize homogenates of eye tissue in a time-course study where either rods were ablated (rod-NTR) or a small subset of bipolar cells were ablated (bipolar-NTR). The responses of the transcriptome were different. Some surprises in the data were that these differences began immediately, which the authors interpret as differences in fate-choice. Some softening of that interpretation (offering alternatives) is suggested below.

Considering the potential for stem-cell-based regenerative therapies in blindness (which is the most likely pioneer for such treatments of other neurodegenerative diseases), the massive data sets produced here are an excellent resource for the community. Their comparative framing (between regeneration paradigms, across time) adds considerably to their anticipated power. It is reasonable to expect that mining these data sets will influence future researchers as they seek to implement plans to therapeutically regenerate cells.

If the work to be repeated today it would consider single cell transcriptomics and allow an additional depth of information, but the data provided here are valuable nonetheless. Some ideas about the manuscript appear below, focused on alternative interpretations the authors might wish to consider.

1) Line 148 & opening of Discussion & closing of Discussion (line 707), regarding the suggestion that the type of cell loss informs the MG stem cell activation via altering *cell-fate* choices. The writing seems to have an unstated assumption that only one population of MG stem cells is present, and only one population is activated regardless of ablation paradigm, and thus the differential outcomes thus must result from differential fate choices. It is important to forthrightly consider an alternative interpretation that multiple populations of stem cells exist, and actually your paradigms are triggering different ones, thereby producing distinct gene abundances at initiation. The wording in abstract (line 49, 57) seems more appropriate, i.e. agnostic to ‘how’ the response is different, and instead identifying that it is different.
 a. Data in Fig 1 (described in lines 227-238) undercut the interpretation that differences in the response are based in cell fate choices, an interpretation that would require the same stem cell population to be activated towards two different states. Instead, what is happening here is that two different stem cell populations are activated. [still very interesting, but must be interpreted with care; and the Rationale that the data give you instructions to replace a particular cell type then seems confounded too]

Author Response: We respectfully disagree with the reviewer on some aspects this point. However, we acknowledge that the similarities in MG stem cell activation between the rod and bipolar paradigms needs to be more prominently emphasized. We have now added a statistical comparison of INL proliferation in rod and bipolar ablated retinas which shows no statistically significant difference in the induction of proliferation in (presumed) MG stem-like cells and MGPCs between the two paradigms (lines 220-236). Thus, our data are consistent with the reviewer’s requirement, that “the same stem cell population…be activated towards two different states”. Clearly, the response of committed rod precursors in the ONL does differ in rod and bipolar ablated retinas (see lines 230-232), but we do not believe this is what the reviewer was referring to with respect to the idea that “multiple populations of [MG] stem cells exist” as ONL precursors are not, by definition, stem cells. We do, however, discuss how differential activation of ONL rod precursors must account for some of the differences we observe (504-506).

We do appreciate that two activated MG subpopulations have been identified based on differential expression of *stat3* and *ascl1a* (Nelson et al., 2012). However, we find no published evidence of MG subtypes dedicated to regenerating specific retinal cell types – as the reviewer suggests. Nor do we think that our data can be used to support the assumption that “what is happening here is that two different stem cell populations are activated”. Nevertheless, we agree that an expanded discussion of this possibility is of interest, especially given data provided in a recent pre-publication using single cell transcriptomics to suggest as many as six different MG subtypes exist in the zebrafish retina (Krylov et al., BioRxiv), and the evidence that selective cell ablation leads to “fate-biased” regenerative responses (see refs in manuscript). This is now included in the Discussion at lines 510-513- “A particularly intriguing possibility is that different subpopulations of MG may exist that are dedicated to the regeneration of specific retinal cell types. Jusuf and colleagues recently presented data consistent with this concept using single cell transcriptomics to suggest as many as six different MG subtypes exist in the zebrafish retina”.

We appreciate that this dataset may not get to the level of demonstrating that the cell lost loss informs cell-fate decisions during regeneration. The data discussed in lines 220-236 shows that rod ablation activated two known stem cell populations (dedicated rod precursors in the ONL and MG/MGPC in the INL). Conversely, and as expected, bipolar loss solely induced MG/MGPC. Unfortunately, the bulk tissue nature of this data limits us from being able to isolate MG changes at each time point, which would be a better way to address this question. We are currently using single-cell multi-omics methods in additional cell-specific paradigms to expand our analysis of this general theme. We anticipate publishing related reports shortly.

In order to more comprehensively discuss the results that this dataset reflects, we have broadened the language to account for these possibilities. Specifically, we changed the wording in line 148 and in the first paragraph of the discussion to acknowledge that two stem cell populations are at play in the rod paradigm (Lines 473-476). Lastly, we changed the final sentence of the first discussion paragraph to "Here, we investigated transcriptomic responses to the selective loss of two “late born” retinal cell types, rod photoreceptors and bipolar interneurons, in an effort to reveal mechanisms that inform and regulate fate-biased regenerative mechanisms in the zebrafish retina” (Lines 493-495).

2) Discussion should be forthright in describing alternative reasons why one might observe differential responses other than just a difference between cell types being ablated.
 a. E.g. a technical artefact could arise if the two lines have different NTR amount or different NTR intracellular-localization, or presence/absence of Gal4-VP16 – any of these could alter the quality of cell death and signals produced even within a given cell type.
 b. The impact of ablating ~hundred cells vs. thousands of cells (in bipolar vs. rod lines) would be expected to be substantial.
 c. Moreover, the random insertion of multiple transgenes differs in the two lines (and likely includes many haplotype differences dragged along with the transgenes in the two lines) such that the genetic background of the compared fish is probably quite different [consider that the standard in mice is to now compare multiple isogenic lines for any transgenic-based phenotype].
 d. It is also possible that the peak of cell death might be offset by several hours in one line vs. the other, due to the Tg or the inherent resilience of the cells, and so the responses would occur in distinct time bins within the circadian cycle.

Author Response: We appreciate the need to further address other potential limitations of the study. We have made the suggested changes in the manuscript to acknowledge these caveats (Lines 506-510).

3) The beginning of the Results has a focus on specificity of the induced cell death (e.g. line 155). The compelling data to challenge this claim must exist within the transcriptomic data sets. E.g. rod opsin and transduction machinery ought to be reduced in abundance for the NTR-rod line compared to controls and compared to the NTR-bipolar line. Bipolar-specific genes should show the opposite pattern. A comment on this seems worth including in this early section of the Results (even if it is to offer the logic and send the reader to later data).
 a. This is alluded to later (line 430) but not with enough detail to serve the purpose requested here. What are some examples of genes and what is their differential abundance? [image resolution in Fig 3b-h is poor, and I cannot read any gene names]

Author Response: To enhance readability of the heat maps in figure 3, we reduced the number of genes listed to the top 25 rather than top 50 and increased the font size. Additionally, we now highlight specific DEGs that validate targeted cell death: selective reduction of *rho* in the rod paradigm (peaking at -0.8 log2 fold change) and *capb5b* loss in the bipolar paradigm (peaking at –0.95 log2 fold change, Lines 266-268). These genes were recently validated as cell-specific markers using single cell transcriptomics (Hoang et, al 2020).

4) Appropriate validation of this transcriptomics approach, considering the presented rationale, would be to show therapeutic potential – i.e. that providing one of the DEG is sufficient to enhance the regeneration. Instead, the authors chose to demonstrate that a DEG, stat3, is required for regeneration. Thus the path to translation has not been clearly established here, though the data is undoubtedly of theoretical interest for that purpose.

Author Response: We thank both reviewers for the push to test additional factors besides *stat3*, with the goal of identifying genes whose disruption led to enhanced rod regeneration (see reviewer one comments for why our expanded analysis was limited to the rod ablation paradigm). We selected 6 new genes for functional tests using our DEG data and related data from two recently published reports. Target genes from our data included *c7b*, *txn, pparpg* and *stat5a/b*. The genes *nfia* and *prdm1a* were originally identified in Hoang et., al 2020 and Lahne et. al, 2020 respectively. Mutation of three of six genes tested led to enhanced rod regeneration (*c7b, txn* and *stat5a/b*). Notably, all three factors are implicated in immune system signaling. Conversely, mutation of *pparg* and *prdm1a* had no effect on rod regeneration. Mutation of *nfia* trended toward a modest enhancement rod regeneration by effect size, but did not reach statistical significance. These results are now mentioned in the abstract (lines 52-54), presented in a new figure 7 (lines 431-456, and discussed at lines 591-596. Combined with our past and recent publications using post-ablation immunosuppression to accelerate rod cell regeneration (White et. al 2017; Emmerich et. al 2023), these results strengthen an argument that the immune system plays a key role in regulating regeneration following selective rod cell loss.

Minor suggestions:
 a) I’m baffled by how the in vivo imaging was accomplished (it is excellent). How does one image through a pigmented eye in a living 11 dpf zebrafish? (roy mutants are still pigmented). I could not find any of this in the Methods or associated Results near description of Figure 1.

Author Response: Thank you! The embryos used in intravital imaging experiments were treated long-term with 1-phenyl 2-thiourea (PTU) to enable imaging through the retina with minimal obstruction. We have now included this detail in the methods section (Lines 612-614) and thank the reviewer for pointing out this lack of detail.

b) where does the CFP in your images come from (Fig 1 legend should indicate this).

Author Response: The CFP expression comes from the *Tg(pax6-DF4:gap43-CFP)q01* line which was noted in the methods but not in the legends for figure 1 or supplemental figure 2. We have now included this detail in these legends (Lines 193-194 and 1056-1058) as well as the methods section (Lines 626-628).

c) Line 35 ‘in’ s/b ‘and’

Author Response: This has been corrected, thank you for identifying this error.

d) Line 70 you implicate immune-related genes, not the immune system itself. It’s only “the immune system” if you accept the over-simplistic assumptions of a KEGG pathway as top-line gene function assignments. “strongly implicated” seems too strong with the adjacent current wording. But I think the authors can be given licence to interpret their data as they prefer, where it is obvious that it is interpretation-not-statement-of-fact.

Author Response: We agree with the reviewer that KEGG, and most pathway analysis tools, produce over-simplified results. In addition, we have reworded the Author Summary to soften the language. From our point of view, the more interesting takeaway was that while many immune system terms were identified, changes in individual immune-related genes were largely unique to one of the two paradigms (lines 67-69, 314-328, and 361-375). With our new data showing that disruption of multiple immune genes (*stat3*, *stat5a/b*, *c7b* and *txn*) had a significant effect on rod regeneration, we feel it is reasonable to suggest a key role for the immune system in rod cell regeneration. To make it clear what key data is leading us to that conclusion, we have reworded the author summary (Lines 67-70).

e) Related to above comment – line 514: is 38% and 44% an impressive number? I think most any DEG data set might show a similar type of abundance of genes related to the immune system. What if you compare the control fish from 6dpf and 11 dpf, is the abundance of these ‘immune-related genes’ <25%? Maybe not an analysis worth doing, but I think you could soften your language: you’ll always find these associations if you go looking for them.

Author Response: While it is true that immune associations are common when analyzing large ‘omics data sets, the language in the manuscript referring to DEG representation in immune signal does not attempt to skew the reader to think the resulting numbers are specifically high or low, rather it plainly states the results (see lines 368-375). That said, in our view, ~40% appeared to be quite a large proportion of the DEGs implicated, especially given that ~27% had been implicated in regeneration as an equally broad term.

f) Line 117, (& 149, 61, 588). “Retinal degenerative diseases typically involve the loss of discrete cell types.” A version of this statement was made earlier too – it seems a bit simple, self-serving, and certainly needs some references and examples. E.g. many blinding disorders involve loss of function mutations that may not (for many years) lead to cell loss (maybe they produce dysmorphic cells). Are there prominent examples of disorders where bipolar cells are suddenly lost? To be more optimistic in my comment, learning how to regenerate a rod photoreceptor is valuable as an imagined therapy for many many forms of blindness, including those where more-than-rods are dying. So it seems like the writing leans on this rationale too hard.

Author Response: We agree that in terms of therapeutic interest the results related to enhanced rod cell regeneration are of high interest to the field and we are pleased to have added new experiments that identified gene disruptions that led to enhanced rod regeneration (Figure 7). The reasoning for our emphasis on retinal diseases linked to selective neuronal cell loss was to set the stage for the value in working with cell-specific ablation models. We have now added disease examples and references to the introduction (Lines 115-117), i.e., retinitis pigmentosa and glaucoma, and added glaucoma to the discussion (Line 468). To acknowledge that cellular dysfunction also has role in disease, we added that to the broader description of retinal diseases (line 624).

g) Line 184 delete ‘selective’. Nothing here demonstrates the ablation is selective (i.e. maybe other cells are killed too).

Author Response: We have removed the word selective (Line 183) to acknowledge the data fail to show that here. However, given that the selectivity of NTR/Mtz-induced cell ablation has been demonstrated in numerous reports across many different targeted cell ablation paradigms (including our initial report, Curado et al. 2007, and our and Hyde lab analyses of NTR-rod ablation paradigms, e.g., Montgomery et al., 2010; Zhang et al., 2021; Sharrock et al., 2022), we feel that this point has been firmly established.

h) Line 197 to allow Figures to be interpretable as stand-alone items, define and/or explain “NTR”.

Author Response: We now define NTR in the figure title (Line 189).
