# Supplementary material for: Transcriptomic comparison of two selective retinal cell ablation paradigms in zebrafish reveals shared and cell-specific regenerative responses
Source: PLoS Genet. 2023 Oct 11;19(10):e1010905. doi: 10.1371/journal.pgen.1010905 (PMC10593236; doi:10.1371/journal.pgen.1010905)
Supplement: Attachment — Submitted filename: 20230727_PlosGeneticsReviewerResponse_ToSUBMIT.docx [file pgen.1010905.s030.docx]

Reviewer's Responses to Questions

**Comments to the Authors:**
**Please note here if the review is uploaded as an attachment.**

Reviewer #1: The authors have sufficiently addressed the previous critiques. However, there are two areas of the manuscript that need to be addressed:

1) “In contrast, bipolar loss led solely to increased INL proliferation” – statistics don’t support this conclusion; only indicate that total proliferation is increased. INL proliferation is listed as 'ns' compared to control

We appreciate that the reviewer caught this, we have now reworded the statement to: “In contrast, bipolar loss only led to increased proliferation when combining INL and ONL counts” (line 225).

2) CRISPants (Figure 7). While the inclusion of this data was recognized as a response to both reviewers, the data do not fully support the Authors' conclusions as presented. The Authors state that "Of the six factors tested (Fig. 7b-c), mutation of three led to enhanced rod regeneration (from least to greatest effect: txn (+70%), stat5a/b +85%), and c7b (+96%), while three had no statistically significant effect (nfia, pparg, prdm1a)" compared to WT animals. While txn, stat5a/b and c7b MUT crispants do seem to have an increase in NTR:YFP compared to WT, other possible explanations need to be addressed. Are rod cell numbers equivalent at the start of the injury paradigm in each mutant condition? Are rod cells more resistant to MTZ-mediated cell death when these specific genes are mutated? Other more nuanced scenarios could also explain the observed results. The Authors should at least examine the effect of each mutation on general retinal development (as they are germline mutations) and acknowledge alternative mechanisms exist that lead to what the Authors interpret as 'enhanced regeneration'.

We agree that there were additional explanations that merited testing to support the results previously included. We examined the effect of each regeneration-enhancing mutant on rod cell development and for any neuroprotective effects induced by ablation. Developmentally, only txn mutation had a mild increase in rod cell fluorescence (+29%) while the other mutants had no effect. Additionally, Mtz-induced ablation brought levels of all 3 mutants, including txn, down to control ablation levels. Increased regeneration following complete ablation demonstrates that the effect on regeneration seen in each case is a true effect. Furthermore, it is possible that similar mechanisms drive increased rod development and regeneration and we plan to investigate this further in future projects.

Reviewer #2: I think this is the best job of addressing Reviewer comments I have seen. Congrats to all the authors on a great contribution!

Thank you!
